# Supplementary material for: Understanding the factors contributing to dengue virus and chikungunya virus seropositivity and seroconversion among children in Kenya
Source: PLoS Negl Trop Dis. 2024 Nov 20;18(11):e0012616. doi: 10.1371/journal.pntd.0012616 (PMC11578454; doi:10.1371/journal.pntd.0012616)
Supplement: S1 Text — (DOCX) [file pntd.0012616.s001.docx]

**Supplementary File**

**Understanding the factors contributing to dengue virus and chikungunya virus**

**seropositivity and seroconversion among children in Kenya**

Amna Tariq MPH, PhD^1*&^, Aslam Khan MD^1*&^, Francis Mutuku PhD^2^, Bryson Ndenga PhD^3^, Donal Bisanzio DVM PhD^4^, Elysse N. Grossi-Soyster MS^1^, Zainab Jembe^5^, Priscilla Maina^5^, Philip Chebii^5^, Charles Ronga^3^, Victoria Okuta^3^, Angelle Desiree LaBeaud MD MS^1^

^1^Department of Pediatrics, Division of Infectious Diseases, Stanford University, Palo Alto, California, USA

^2^Department of Environment and Health Sciences, Technical University of Mombasa, Mombasa, Kenya

^3^Centre for Global Health Research, Kenya, Medical Research Institute, Kisumu, Kenya

^4^RTI International, Washington, D.C., USA

^5^Vector borne Disease control Unit, Msambweni County Referral hospital, Msambweni, Kenya

[*atariq1@stanford.edu](mailto:*atariq1@stanford.edu)

^&^Both authors contributed equally to this study

| Item | Categories |
| --- | --- |
| House floor material | Dirt, wood, tile, cement |
| House roof material | Corrugated iron, natural materials, roofing tiles |
| Type of toilet in house | Pit latrine, VIP, none, flush |
| House water supply | Piped public, river canal, public well, piped house, borehole, rain, dam pond, borehole pump |
| Light source in the house | Lantern, solar, electricity |
| Livestock in the house | Yes/no |
| Durable assets | Radio, TV, car, bicycle and telephone |

Table A: List of items and categories used to construct the wealth index.

| Year | DENV seroprevalence | CHIKV seroprevalence |
| --- | --- | --- |
| 2014 | 10 (0.29%) | 23 (0.66%) |
| 2015 | 38 (1.10%) | 85 (2.46%) |
| 2016 | 44 (1.27%) | 71 (2.06%) |
| 2017 | 68 (1.97%) | 112 (3.25%) |
| 2018 | 21 (0.60%) | 29 (0.85%) |

Table B. DENV and CHIKV seroprevalence in Kenya by year.

| Predictor | DENV seropositivity  OR (95% CI) | CHIKV seropositivity  OR (95% CI) |
| --- | --- | --- |
| Age | **1.16 (1.10, 1.22)**** | **1.10 (1.06, 1.14)***** |
| Gender  Male | 0.43 (0.84, 1.52) | 1.25 (0.99, 1.57) |
| Site  West | **0.51 (0.37, 0.69)***** | **2.77 (2.16, 3.57)***** |
| Area density  More densely populated “urban” site | **0.49 (0.36, 0.67)***** | **0.22 (0.17, 0.29)***** |
| Crowding | **0.69 (0.47, 0.99)** | **0.59 (0.43, 0.78)***** |
| Presence of water buckets | **1.48 (1.09, 2.01)***** | **1.51 (1.20, 1.91)***** |
| Wealth index | **0.82 (0.72, 0.93)**** | **0.82 (0.74, 0.91)***** |
| Child travel^a^ | 1.20 (0.88, 1.62) | 1.09 (0.86, 1.39) |
| Child outdoor time | 1.70 (0.95, 3.37) | 1.02 (0.71, 1.53) |
| Presence of trash in compound | 0.95 (0.72, 1.40) | **1.85 (1.46, 2.34)***** |
| Presence of window screens in house | 1.10 (0.81, 1.48) | **0.72 (0.48, 0.79)***** |
| Mosquito index^b^ | 0.67 (0.35, 1.16) | 1.42 (0.98, 2.00) |

Table C: Univariable logistic regression analysis of the risk factors associated with DENV and CHIKV seroprevalence.

*Indicates a statistically significant p-value, 0.05*, 0.01**, 0.001***

^a^ Travel indicates the travel of a child more than 10km in the last six months

^b^ Mosquito index comprises of the usage of mosquito coil, mosquito repellent or mosquito spray by the children

| Predictor | DENV seroconversion  (95% CI) | CHIKV seroconversion  (95% CI) |
| --- | --- | --- |
| Age | **1.10 (1.03, 1.17)**** | 1.02 (0.98, 1.07) |
| Gender  Male | 1.35 (0.92, 2.00) | 1.23 (0.90, 1.67) |
| Site  West | 0.81 (0.54, 1.19) | **1.74 (1.28, 2.40)***** |
| Area density  More densely populated “urban” site | **0.42 (0.28, 0.63)***** | **0.22 (0.15, 0.31)***** |
| Crowding | **0.57 (0.34, 0.91)*** | **0.51 (0.34, 0.75)**** |
| Presence of water buckets in house | **2.31 (1.54, 3.53)***** | **1.96 (1.43, 2.71)***** |
| Wealth index | 0.86 (0.72, 1.01) | **0.80 (0.70, 0.91)**** |
| Child travel^a^ | **1.61 (1.09, 2.37)*** | 1.35 (0.99, 1.84) |
| Child outdoor time | 1.36 (0.72, 2.93) | 0.99 (0.62, 1.68) |
| Presence of trash in compound | 1.23 (0.80, 1.83) | **1.83 (1.34, 2.49)***** |
| Presence of window screens | 1.04 (0.70, 1.52) | **0.72 (0.52, 0.99)*** |
| Mosquito index^b^ | 0.88 (0.41, 1.65) | 0.90 (0.50, 1.49) |

Table D: Univariable logistic regression analysis of the risk factors associated with DENV and CHIKV seroconversion.

*Indicates a statistically significant p-value , 0.05*, 0.01**, 0.001***

^a^ Travel indicates the travel of a child more than 10km in the last six months

^b^ Mosquito index comprises of the usage of mosquito coil, mosquito repellent or mosquito spray by the children
