# Supplementary material for: Understanding the factors contributing to dengue virus and chikungunya virus seropositivity and seroconversion among children in Kenya
Source: PLoS Negl Trop Dis. 2024 Nov 20;18(11):e0012616. doi: 10.1371/journal.pntd.0012616 (PMC11578454; doi:10.1371/journal.pntd.0012616)
Supplement: Attachment — Submitted filename: Response_to_reviewers_may13_2024_at_AK_at_final.docx [file pntd.0012616.s002.docx]

**Reviewer's Responses to Questions**


We thank our reviewers for their time and efforts in reviewing the manuscript and providing constructive feedback to our team. The comments from the reviewers have been addressed in the manuscript and below we provide a point-by-point response. We believe the reviewer suggestions have significantly improved the manuscript.

**Methods

Reviewer #1: (No Response)

Reviewer #2: The statistical methods that were used in determining results, such as logistic regression and Kaplan-Meier analysis, were not clearly described.**

**Response:** We have explained the logistic regression using the Generalized linear mixed models and the Kaplan-Meier analysis in more detail in the methods section (lines 194-220).
**--------------------

Results

Reviewer #1: (No Response)

Reviewer #2: They attempted to identify the predictors of DENV and CHIKV seroprevalence and seroconversion and describe the temporal patterns of DENV and CHIKV seroconversion. However, the authors did not adequately describe the rationale, significance, and inclusion and exclusion criteria of the study.**

**Response:**

We have included the rationale (lines 99-106), significance (lines 109-112), and inclusion and exclusion criteria of the study (lines 132-135) in the methods and introduction section.

**--------------------

Conclusions


Reviewer #1: (No Response)

Reviewer #2: The authors should clearly demonstrate the novel findings of their study in the body of literature. This could be achieved by highlighting how their study adds to existing literature and identifying any new or significant findings.**

**Response:** We have added the significant findings and how this study adds to the existing literature in the discussion and conclusion sections (lines 437-444, 462-478).
 **--------------------

Editorial and Data Presentation Modifications?


Use this section for editorial suggestions as well as relatively minor modifications of existing data that would enhance clarity. If the only modifications needed are minor and/or editorial, you may wish to recommend “Minor Revision” or “Accept”.

Reviewer #1: (No Response)

Reviewer #2: (No Response)

--------------------

Summary and General Comments


Use this section to provide overall comments, discuss strengths/weaknesses of the study, novelty, significance, general execution and scholarship. You may also include additional comments for the author, including concerns about dual publication, research ethics, or publication ethics. If requesting major revision, please articulate the new experiments that are needed.

Reviewer #1: In this prospective cohort study, authors aim to identify non-biological risk factors associated with DENV/CHIKV infection in Kenyan children, diseases causing less morbidity/mortality in Kenya compared to other endemic countries. It is a novel/significant study given the uncharacterised behaviour of these infections in sub-Saharan countries and also a major effort considering the study was conducted for five years.

Unfortunately, results are unnecessarily extended, making repetitive and uninformative sections that are closely related (e.g., seroprevalence and incidence) or don't have a fundamental implication for policy-making. What is the usefulness of exploring seroprevalence and seroconversion separately? Despite these informing different phenomena (e.g., symptomatic vs asymptomatic infection), how is this data expected to help policy-making or public health? Too many tables and a massive amount of figures make the manuscript overwhelming and lose perspective. Authors should focus on one aspect, justifying the selection by evidence, and remake the result section to make it friendly and catchy.**

**Response:**

We have included across the manuscript in different sections how these results will inform the public health policy and strategies for dengue and chikungunya virus control. Moreover, it has also been clarified in the manuscript why seroprevalence and seroconversion were explored separately. The result section has been rearranged by moving some tables to the supplementary file and combining the Kaplan-Meier curve figures together to make it more reader friendly.

**Major Issues: - As exposed in the previous section, results are unnecessarily long and should be restricted to one relevant and major aspect: seroprevalence or seroconversion. Additional data could be included as supplementary in case readers are interested in specific details.**

**Response:**

The purpose of the study is to assess the risk factors associated with both the seroprevalence and seroconversion of DENV and CHIKV in the pediatric cohort followed over five years. Seroprevalence provides us an understanding of the disease burden in the population and the seroconversion rates help quantify the number of children becoming positive for CHIKV or DENV between any two time points in the study, providing us with an understanding of active ongoing CHIKV and DENV infection among our cohort (lines 101-107).

**- Statistical analysis: I agree with the authors in using PCA to reduce multiple variables and create specific scores. However, these artificial new variables need to be validated against an independent variable to confirm new scores are solid and actually capturing what is expected. Just as an example, if there's a correlation between the SES and yearly/weekly income, there's solid evidence of the usefulness of the new scores.**

**Response:**

Unfortunately, we did not have information on the income level of the cohort and the wealth index has been created by combining various predictors that depict wealth status of the participants such as the presence of motorcycle, bike, tv, radio in the house and the kind of flooring, toilet, windows and roofing in the house. As such when correlation is checked between the different predictors and the wealth index, we observe the following correlation matrix, emphasizing the importance of the use of PCA to create the wealth index.

**Minor Issues: - In Table 3, the gender male is reported as significant, although the CI is 0.99 - 1.57. Please be more careful when making tables and report results.

- Tables are enumerated incorrectly from Table 4 onwards.**

**Response:**

The manuscript has been checked and corrected for all enumerations of tables, figures, and significance of predictors.
 **Reviewer #2: The authors undertook a prospective study on factors associated with seroprevalence and seroconversion of DENV and CHIKV among children in Kenya. They attempted to identify the predictors of DENV and CHIKV seroprevalence and seroconversion and describe the temporal patterns of DENV and CHIKV seroconversion. However, the authors did not adequately describe the rationale, significance, and inclusion and exclusion criteria of the study. The statistical methods that were used in determining results, such as logistic regression and Kaplan-Meier analysis, were also not clearly described. It is therefore recommended that the authors revise the introduction and methods sections to fully elucidate their approach. Additionally, the study had minor grammatical issues. In addition, authors should consider modifying their manuscript title. Lastly, and most importantly, the authors should clearly demonstrate the novel findings of their study in the body of literature. This could be achieved by highlighting how their study adds to existing literature and identifying any new or significant findings.**

**Response:**

We have included an explicit explanation on the rationale (lines 101-106), significance (lines 108-118), and inclusion and exclusion criteria of the study (lines 131-135). Moreover, as per reviewers’ suggestion, added explanation has been included in the methods to describe the statistical analysis. We have modified the introduction, title, methods and discussion taking into account reviewers’ comments and added the novelty of the study section (lines 44-443) in the manuscript and how it adds to literature (lines 458-475).
