# Supplementary material for: Understanding the factors contributing to dengue virus and chikungunya virus seropositivity and seroconversion among children in Kenya
Source: PLoS Negl Trop Dis. 2024 Nov 20;18(11):e0012616. doi: 10.1371/journal.pntd.0012616 (PMC11578454; doi:10.1371/journal.pntd.0012616)
Supplement: Attachment — Submitted filename: Response_to_Reviewer_sept13_2024_final.docx [file pntd.0012616.s003.docx]

**Reviewer's Responses to Questions**

Thank you for your comments. Below we provide point by point response to each comment.
 **-Are the objectives of the study clearly articulated with a clear testable hypothesis stated?
-Is the study design appropriate to address the stated objectives?
-Is the population clearly described and appropriate for the hypothesis being tested?
-Is the sample size sufficient to ensure adequate power to address the hypothesis being tested?
-Were correct statistical analysis used to support conclusions?
-Are there concerns about ethical or regulatory requirements being met?**

**1. Lines 124-125: There is a lack of clarity in the methods section regarding the definitions and criteria for seropositivity and seroconversion for DENV. 
   a. Within this definition issue, should individuals who are already seropositive (IgG+ at baseline) be excluded from the follow-up when mapping seroconversion?
   b. The same question (1a) applies to CHIKV cases.**

Response: We have defined seropositivity and seroconversion for DENV and CHIKV cases in lines 130-134 and defined the criteria for seroconversion In lines 145-148.

**2. Line 267: It is unclear whether the univariate analysis of variables against seropositivity accounted for data across the 5-year follow-up period. Since variables such as wealth index, population, and crowding index are not constant and likely change during the study period, how did the authors account for these changes in their analysis?**

Response: For each of the predictors included in the univariable and multivariable analysis, the most complete responses were recorded at the baseline. For each of the follow up visits, majority of the responses to the questions were incomplete or missing and when we looked at heterogeneity across responses over time, there were no marked changes among our population. Therefore, for any given predictor, if the study participant answered a yes in response to that predictor over any of the visits or the baseline, the response was recorded as a “yes” for analysis purposes (Lines 189-196)

**3. There is a repeated lack of clarity regarding the definitions and cutoffs for various variables. For instance, the definition of the "type" of water container is unclear, the cutoff for 10 km in line 293 is unclear, the crowding index definition is unclear, and the definition of "outdoor time" is unclear. Additionally, it is not clear which specific water containers were considered and their relevance to the study's aim. If the study's focus is on mosquito breeding, it should include information on how long containers need to be left unused to be optimal for breeding. What happens to a bucket that is used 5 times daily and kept open? Does this bucket increase mosquito breeding chances? The contextual information seems to be missing.**

Response: We have clarified the definitions of the type of water container (lines 218-221), travel (lines 231-232), and crowding index (lines 212-216) in the manuscript. There was no information collected on how often the water buckets were emptied or left unused, however, these water containers including water buckets, barrels and tires were most commonly found to be breeding the mosquitoes.

**4. Line 238: It is suggested to refer to the method for the “mosquito index.” However, it is unclear where this index is defined in the methods section.**

Response: Mosquito index has been added in the methods section, lines 216-218.

**5. Line 190: It is written that the “crowding index was defined as the number of residents living in the household divided by the number of rooms in the house.” What happens if a household has larger rooms but fewer rooms overall? How do you account for density in such cases?**

Response: The only information collected on house rooms was the number of rooms. The size of the rooms was not included in the study questionnaire, therefore we do not have any data to account for the density.

**6. Line 203: The definition of “outdoor time” is missing. Since the study includes both housebound infants and school-going children, the latter will bias the outdoor time regardless, as infants have less mobility**.

Response: We have clarified the definition of outdoor time in lines 233-235. The children of age less than 2 years old were 92 among the whole cohort of 3445 study participants and 73 of them reported to have outdoor time.

**Results-**

**Reviewer #3: 1. Table 1: Outdoor Time: Of the 322 individuals (9.3%) marked as having “No” outdoor time, what age group do they represent? Is this group primarily composed of infants?**

Response: Infants are not the only age group with no outdoor time. Below we provide the distribution of the outdoor time with age as follows (154 missing observations due to missing data on age):

| Age | Missing | No outdoor time | Yes outdoor time | Percentage of participants with no outdoor time (%) |
| --- | --- | --- | --- | --- |
| 1 | 2 | 0 | 3 | 0 |
| 2 | 4 | 13 | 70 | 14.9 |
| 3 | 38 | 35 | 192 | 13.2 |
| 4 | 71 | 29 | 210 | 9.35 |
| 5 | 70 | 32 | 223 | 9.84 |
| 6 | 65 | 33 | 232 | 10 |
| 7 | 69 | 26 | 220 | 8.25 |
| 8 | 66 | 31 | 213 | 10 |
| 9 | 68 | 31 | 211 | 10 |
| 10 | 60 | 29 | 200 | 10.03 |
| 11 | 57 | 23 | 189 | 8.55 |
| 12 | 54 | 28 | 175 | 10.89 |
| 13 | 54 | 8 | 98 | 5 |
| 14 | 21 | 4 | 14 | 10.25 |
| 15 | 8 | 0 | 6 | 0 |
| 16 | 6 | 0 | 0 | 0 |

**2. Line 256: "DENV seroprevalence exhibited an increase from 2014-2017 with a decline in 2018." Can you please indicate which figure or table this statement is referring to?**

Response: We have added the table (Supplementary file Table 2) and cited it in the paper to clarify the seroprevalence for DENV and CHIKV per year.

**3. Line 282: The author states, "…..decreased odds of DENV seropositivity" and relates this to protection in Abstract Line 43. Clarification is needed on the premise that the decrease in odds ratio (OR) for seropositivity is related to protection against DENV.**

Response: We have changed the language in the manuscript to avoid any confusion, line 46.

**Conclusions**

**Reviewer #3: 1. Are the conclusions supported by the data presented?**

**Answer: The conclusions are not fully supported by the data presented. The reviewer has identified several areas where the methods and interpretations lack clarity or rigor, which undermines the confidence in the conclusions drawn from the study. Specific concerns include the definitions and criteria for seropositivity and seroconversion, the handling of changing variables over the study period, and the contextual relevance of certain variables.**

Response: We have clarified the definitions of seropositivity, seroconversion, the criteria for seroconversion analysis, and explained how we handle the data for the purpose of this analysis. There was no marked change in predictors over time, and for the predictors such as wealth index, mosquito index and, crowding index, there was no change in the calculation at each time point.

2**. Are the limitations of analysis clearly described?
Answer: The limitations of the analysis are not clearly described. The reviewer points out multiple methodological issues that need to be addressed, such as the unclear definitions of key variables, how changes in socio-economic factors were accounted for, and the lack of clarity in the analysis of the univariate and multivariate models. These limitations need to be explicitly stated and discussed in the study.**

Response: We have explained in the limitations, why we combine the responses at baseline and follow-up visits. There were no marked changes in responses over time among the population, so the indices created such as the wealth index, crowding and mosquito index do not change over time. Definitions of the key predictors have been clarified.

**3. Do the authors discuss how these data can be helpful to advance our understanding of the topic under study?
Answer: The abstract does not sufficiently discuss how the data can advance understanding of the topic. While the study provides data on seropositivity and seroconversion rates and identifies some risk factors, the lack of methodological clarity and rigorous interpretation limits the extent to which these findings can be considered robust contributions to the field. The authors need to more clearly articulate how their findings contribute to existing knowledge and what implications they have for future research and public health interventions.**

Response: We have included in the abstract how this study can advance understanding of the topic (lines 58-63). We have expanded on the findings and how they contribute to existing knowledge, future research and, public health interventions in lines 512-525.

**4. Is public health relevance addressed?
Answer: The public health relevance is partially addressed. The study aims to identify risk factors for DENV and CHIKV seropositivity and seroconversion, which has clear public health implications. However, due to the identified issues with the methods and interpretations, the practical applications and significance of the findings are not fully clear. The authors need to better highlight how their findings can inform public health strategies and interventions, especially in the context of vector control and disease prevention in endemic areas.**

Response: We have expanded on the public health strategies and interventions (lines 474-480).

--------------------

**Reviewer #3: Dengue virus (DENV) and chikungunya virus (CHIKV) are significant causes of febrile illness among children in Kenya. This study investigates the relationships between various household, socio-economic, demographic, and behavioral risk factors and the seropositivity and seroconversion of DENV and CHIKV in four settlements in Kenya. A pediatric cohort of 3,445 children was followed prospectively from 2014 to 2018. Temporal patterns of seroconversion were described using Kaplan-Meier curves, and logistic regression with generalized linear mixed models identified potential exposure risk factors.

Reviewer Comments Summary
1. Definitions and Criteria (Lines 124-125):
   - Clarification needed on definitions and criteria for seropositivity and seroconversion for DENV.
   - Should seropositive individuals at baseline be excluded from follow-up when mapping seroconversion?
   - The same question applies to CHIKV cases.**

Response: The definitions have been clarified in lines 130-134. And it has been explicitly stated in the manuscript that for the purpose of DENV and CHIKV seroconversion, participants who were DENV or CHIKV positive at the baseline were excluded from the study.

**2. Univariate Analysis (Line 267):
   - Unclear if univariate analysis accounted for data across the 5-year follow-up period.
   - Variables such as wealth index, population, and crowding index likely changed during the study. How were these changes accounted for in the analysis?**

Response: We have explained in lines 189-196 and lines 230-236 how we accounted for the predictors in the analysis. **3. Variable Definitions and Cutoffs:
   - Repeated lack of clarity on definitions and cutoffs for various variables.
   - Specific concerns about the "type" of water container, the cutoff for 10 km, the crowding index definition, and the definition of "outdoor time."
   - Need clarification on the relevance of specific water containers to the study's aim, particularly regarding mosquito breeding.**

Response**:** We have clarified the water containers used in the analysis (lines 218-220), definition of the crowding index in lines 212-216 and, outdoor time in lines 232-233.
 **4. Mosquito Index (Line 238):
   - Reference to "mosquito index" is unclear in the methods section.**

Response: We have clarified the mosquito index in the methods section (lines 216-218)
 **5. Crowding Index (Line 190):
   - Definition provided but unclear how density is accounted for in households with larger rooms but fewer rooms overall.**

Response: No information on the size of the rooms is available so density can not be accounted for.
 **6. Outdoor Time (Line 203):
   - Missing definition of "outdoor time."
   - Bias potential between housebound infants and school-going children, as infants have less mobility.**

Response: We have added the definition of outdoor time (lines 233-235). A total of 73 participants under the age of 2 years declared to have some outdoor time during the day.

**Specific Results Clarifications
1. Table 1: Outdoor Time:
   - For the 322 individuals (9.3%) marked as having “No” outdoor time, what age group do they represent? Is this group primarily composed of infants?**

Response: The distribution of the outdoor time with age is given as follows:

| Age | Missing | No outdoor time | Yes outdoor time | Percentage of participants with no outdoor time (%) |
| --- | --- | --- | --- | --- |
| 1 | 2 | 0 | 3 | 0 |
| 2 | 4 | 13 | 70 | 14.9 |
| 3 | 38 | 35 | 192 | 13.2 |
| 4 | 71 | 29 | 210 | 9.35 |
| 5 | 70 | 32 | 223 | 9.84 |
| 6 | 65 | 33 | 232 | 10 |
| 7 | 69 | 26 | 220 | 8.25 |
| 8 | 66 | 31 | 213 | 10 |
| 9 | 68 | 31 | 211 | 10 |
| 10 | 60 | 29 | 200 | 10.03 |
| 11 | 57 | 23 | 189 | 8.55 |
| 12 | 54 | 28 | 175 | 10.89 |
| 13 | 54 | 8 | 98 | 5 |
| 14 | 21 | 4 | 14 | 10.25 |
| 15 | 8 | 0 | 6 | 0 |
| 16 | 6 | 0 | 0 | 0 |

Infants are not the only age group with no outdoor time. 14.9% infants declined having any outdoor time. The age on 154 (4.4%) study participants is missing in the data.

**2. Seroprevalence Trend (Line 256):
   - "DENV seroprevalence exhibited an increase from 2014-2017 with a decline in 2018." Indicate which figure or table this statement refers to.**

Response: We have included a table in supplementary file, Table 2. **3. Decreased Odds of Seropositivity (Line 282):
   - Clarification needed on how the decreased odds ratio (OR) for seropositivity relates to protection against DENV.**

Response: We have changed the language in the manuscript to avoid any confusion (lines 46-47).
--------------------
